# Blocking of Caspases Exerts Anti-Inflammatory Effects on Periodontal Cells

**DOI:** 10.3390/life12071045

**Published:** 2022-07-13

**Authors:** Layla Panahipour, Lara Cristina Cunha Cervantes, Azarakhsh Oladzad Abbasabadi, Mariane Beatriz Sordi, Zahra Kargarpour, Reinhard Gruber

**Affiliations:** 1 Department of Oral Biology, University Clinic of Dentistry, Medical University of Vienna, Sensengasse 2a, 1090 Vienna, Austria; layla.panahipour@meduniwien.ac.at (L.P.); lara.cervantes@unesp.br (L.C.C.C.); azarakhsh.oladzadabbasbadi@meduniwien.ac.at (A.O.A.); mariane.sordi@kcl.ac.uk (M.B.S.); zahra.kargarpooresfahani@meduniwien.ac.at (Z.K.); 2 Department of Diagnosis and Surgery, School of Dentistry, São Paulo State University (UNESP), Araçatuba, Sao Paulo 16015-050, Brazil; 3 Centre for Research on Dental Implants (CEPID), Department of Dentistry, Federal University of Santa Catarina (UFSC), Florianopolis 88040-900, Brazil; 4 Department of Periodontology, School of Dental Medicine, University of Bern, Freiburgstrasse 7, 3010 Bern, Switzerland; 5 Austrian Cluster for Tissue Regeneration, Donaueschingenstraße 13, 1200 Vienna, Austria

**Keywords:** caspase, fibroblast, inflammation, in vitro, macrophage, periodontitis

## Abstract

Periodontitis is an inflammatory process that is associated with caspase activity. Caspases could thus become molecular targets for the modulation of the inflammatory response to harmful factors, such as lipopolysaccharides (LPS) and TNFα. Here, the impact of the pan-caspase inhibitor Z-VAD-FMK (carbobenzoxy-valyl-alanyl-aspartyl-[O-methyl]-fluoro-methyl ketone) on the modulation of the LPS-induced inflammatory response of murine RAW 264.7 cells and primary macrophages was examined. Moreover, the inflammatory responses of human gingival fibroblasts, HSC2 oral squamous carcinoma cells and murine ST2 mesenchymal fibroblasts when exposed to TNFα were studied. Data showed that Z-VAD-FMK significantly lowered the inflammatory response of RAW 264.7 cells and primary macrophages, as indicated by the expression of IL1 and IL6. In murine ST2 mesenchymal fibroblasts, the TNFα-induced expression of CCL2 and CCL5 was significantly reduced. In human gingival fibroblasts and HSC2 cells, Z-VAD-FMK considerably reduced the TNFα-induced expression of CXCL8 and CXCL10. These findings suggest that pharmacological blocking of caspases in an inflammatory environment lowers the expression of cytokines and chemokines in periodontal cells.

## 1. Introduction

Periodontitis is a chronic inflammatory disease in which the virulence factors released by the surrounding biofilm are constantly challenging the innate defense system [1,2]. Virulence factors, including lipopolysaccharides (LPSs), activate the patter-recognition receptors of macrophages and provoke the expression of cytokines, with TNFα being a major inflammatory player [3]. For instance, the inflammatory response of fibroblasts is initiated by TNFα [4]. Moreover, oral epithelial cells are susceptive to TNFα [5]. TNFα is thus part of the self-amplification inflammatory process characterized by the release of other inflammatory mediators, such as CXCL2 [6], CXCL8 [7,8] and CXCL10 [9,10]. Chronic inflammation culminates in the destruction and, hence, loss of periodontal tissue integrity [11,12,13,14]. Apart from eliminating the biofilm, pharmacological strategies targeting the molecular pathways of cell death and the expression of inflammatory mediators are feasible for combatting periodontitis.

Caspases represent a family of proteolytic enzymes that play essential roles in programmed cell death [15]. These caspases act by triggering cell death via caspase-3, caspase-1/11 and caspase-8 elicit pyroptosis [16,17] and necroptosis [18], respectively. Apoptosis, pyroptosis and necroptosis are functionally activated by an inflammatory environment, and the main pathways are the LPS-TLR and the TNF-mediated signaling pathways. It is the activation and blocking of the respective caspases, particularly caspase-8, that direct the cell death pathway [19]. The compound Z-VAD-FMK (Z-VAD), a pan-caspase inhibitor, not only prevents apoptosis in various cell types but can additionally inactivate necroptosis by inhibiting caspase-8 [20]. In chronic periodontitis, apoptosis [21], pyroptosis [22,23] and necroptosis [24,25] are active and can therefore affect periodontal tissue integrity. It might be interesting to determine whether Z-VAD blocks the secretion of inflammatory cytokines of gingival fibroblasts and oral epithelial cells, similarly to what is known for macrophages [26,27,28].

Pharmacological application of Z-VAD reduced the mortality rate of mice treated with LPSs and blocked the secretion of inflammatory cytokines in macrophages [26,27,28]. Recently, we have shown that inhibition of apoptosis by Z-VAD attenuates shape changes in the alveolar ridge following tooth extraction in rodents [29]. Furthermore, in rats, pan-caspase inhibitors reduced the dysfunction of acute renal failure [30], the progression of experimental glomerulonephritis [31], brain damage after subdural hematoma [32] and the number of endotoxin cellular events in the liver [33]. This accumulating evidence suggests that pan-caspase inhibitors have anti-inflammatory properties. Thus, the ground for the tenor of all multidisciplinary research involving pan-caspase inhibitors is the same; explicitly, it reduces inflammation and, consequently, tissue damage. However, whether this beneficial effect of pan-caspase inhibitors is applicable to defend against the chronic inflammation of periodontitis and periimplantitis has not been studied [24,25]. This is a proof-of-principle question since the clinical use of caspase inhibitors has already been approved as effective and safe [34].

Considering this premise, we show here that, consistent with reports on LPS-stimulated macrophages [26,27,28], Z-VAD reduces the secretion of inflammatory cytokines. Based on this model, we report that Z-VAD attenuates the TNFα-induced expression of inflammatory mediators CXCL8 and CXCL10 in gingival fibroblast and HSC2 oral epithelial cells.

## 2. Material and Methods

### 2.1. Murine ST2 Cells, Human Gingival Fibroblasts and HSC2 Oral Squamous Carcinoma Cells

The ST2 mesenchymal stromal cell line was originally isolated from mouse bone marrow (Riken Cell Bank, Tsukuba, Japan). Human gingiva was harvested from extracted wisdom teeth from patients who had given informed and written consent. Approval was obtained from the Ethics Committee of the Medical University of Vienna (EK NR 631/2007). Gingival tissues were washed in phosphate-buffered saline (PBS) to dilute the oral bacterial flora. Following that, the tissues were cut into small pieces of between 1 and 2 mm^2^ using a #10 surgical blade. The medium used for culture and expansion of these cells consisted of standard alpha Minimum Essential Medium Eagle (αMEM), supplemented with 10% fetal calf serum (Bio&Sell GmbH, Nuremberg, Germany) and 1% antibiotics (Sigma Aldrich, St. Louis, MO, USA). The medium was changed every 2 to 3 days. A total of three strains of fibroblasts were generated from explant cultures and fewer than five passages were used for the experiments. The oral squamous carcinoma cell line HSC2 was obtained from the Health Science Research Resources Bank (Sennan, Japan).

The cells were expanded in growth Dulbecco’s Modified Eagle’s Medium (DMEM, Sigma Aldrich, St. Louis, MO, USA) supplemented with 10% fetal calf serum (Bio&Sell GmbH, Nuremberg, Germany) and 1% antibiotics (Sigma Aldrich, St. Louis, MO, USA), then seeded at 3 × 10^5^ cells/cm^2^ into 24-well plates under standard conditions at 37 °C, 5% CO_2_ and 95% humidity. The following day, cells were exposed to 20 µM Z-VAD-FMK (Selleckchem, Houston, TX, USA) for half an hour before adding 20 ng/mL mouse or human TNFα (ProSpec-Tany TechnoGene Ltd., Rehovot, Israel). After 6 h, gene expression analysis was performed from RNA and supernatant was collected for immunoassays.

### 2.2. Isolation and Culture of Murine Bone Marrow-Derived Macrophages and RAW264.7 Cells

Bone marrow cells were collected from the femora and tibiae of Balb/c mice aged 6–8 weeks old. After euthanizing the mouse, the femora and tibiae area with 70% ethanol were soaked. Following that, the skin and muscles of the femora and tibiae were removed. The bones were separated from the joints and flushed with DMEM using a 23 G needle. Bone marrow cells were passed through a 100 µm cell strainer (pluriSelect Life Science, Leipzig, Germany). After seeding at 3 × 10^6^ cells/cm^2^ into 12-well plates, cells were grown for 7 days in DMEM supplemented with 10% fetal bovine serum, 1% antibiotics (all Invitrogen, Grand Island, NY) and 30 ng/mL M-CSF (Prospec, Ness-Ziona, Israel). RAW 264.7 macrophage-like cells were expanded in growth medium and seeded 1 × 10^6^ cells/cm^2^ into 12-well plates under standard conditions at 37 °C, 5% CO_2_ and 95% humidity. The following day, cells were exposed to 20 µM Z-VAD-FMK (Selleckchem, Houston, TX, USA) for half an hour before adding 100 ng/mL LPSs from *Escherichia coli 055:B5* (ProSpec-Tany TechnoGene Ltd., Rehovot, Israel). After 6 h, gene expression analysis was performed from the collected cell RNA, and supernatant was collected for immunoassays.

### 2.3. qRT-PCR Analysis and Immunoassay

Total RNA was isolated with the ExtractMe total RNA kit (Blirt S.A., Gdańsk, Poland) and followed by reverse transcription (LabQ, Labconsulting, Vienna, Austria) and polymerase chain reaction (LabQ, Labconsulting, Vienna, Austria) on a CFX Connect Real-Time PCR Detection System (Bio-Rad, Hercules, CA). The primer sequences were: mIL1-F TTGGTTAAATGACCTGCAACA, mIL1_R GAGCGCTCACGAACAGTTG; mIL6-F GCTACCAAACTGGATATAATCAGGA, mIL6-R CCAGGTAGCTATGGTACTCCAGAA; mCOX2-F CAGACAACATAAAACTGCGCCTT, mCOX2-R GATACACCTCTCCACCAATGACC; mCCL2-F GCTACAAGAGGATCACCAGCAG, mCCL2-R GTCTGGACCCATTCCTTCTTGG; mCCL5-F CCTGCTGCTTTGCCTACCTC, mCCL5-R ACACACTTGGCGGTTCCTTC; mGAPDH-F AACTTTGGCATTGTGGAAGG, mGAPDH-R GGATGCAGGGATGATGTTCT; hCXCL8-F AACTTCTCCACAACCCTCTG, hCXCL8-R TTGGCAGCCTTCCTGATTTC; hCXCL10-F TGCCATTCTGATTTGCTGCC, hCXCL10-R TGCAGGTACAGCGTACAGTT; hGAPDH-F AAGCCACATCGCTCAGACAC, hGAPDH-R GCCCAATACGACCAAATCC. The mRNA levels were calculated by normalizing to the housekeeping gene GAPDH using the ^ΔΔ^Ct method after exponential expression transformation. The immunoassays for human IL8 (DY208, R&D Systems, Minneapolis, MN, USA) and mouse IL6 (DY406, R&D Systems, Minneapolis, MN, USA) were performed with the cells’ supernatant.

### 2.4. Fluorescence Microscopy

Cells were seeded into Millicell^®^ EZ slides (Merck KGaA, Darmstadt, Germany), subjected to the above-mentioned stimulation and then fixed with 4.0% formaldehyde at room temperature for 20 min. After washing, cells were permeabilized with 0.1% of Triton X-100 in PBS for 5 min and rinsed with PBS at room temperature three times. Phalloidin-Fluor (Santa Cruz Biotechnology, Inc., Dallas, TX, USA) at a 1:1000 dilution was added, and the cells were mounted with Fluoromount-G Mounting Medium with DAPI (Invitrogen, Thermo Fischer, Waltham, MA, USA). Images were taken using a Revolve fluorescent microscope (Echo, San Diego, CA, USA) with filter blocks for FITC and DAPI.

### 2.5. Caspase-8 Activity Assay

Cells were plated at a density of 1 × 10^4^ cells/cm^2^ in a 96-well plate and subjected to overnight stimulation with either LPS or TNFα in the presence or absence of Z-VAD. After stimulation, the equivalent media volume of Caspase-Glo^®^ 8 Reagent (Promega, Madison, WI, USA) was added and the luminescence of each sample was measured.

### 2.6. Statistical Analysis

All experiments were repeated at least three times. Data from individual experiments are shown as dot-blots. If not otherwise indicated, data are described as fold-changes compared to controls. Controls were unstimulated cells in serum-free DMEM. An inflammatory response was defined as a minimum 10-fold increase in the expression of inflammatory cytokines. Statistical analysis was based on a paired *t*-test and Friedman test. The *p*-values are indicated in the respective figures. Analyses were performed using Prism v8 (GraphPad Software, La Jolla, CA, USA). Significance was set at *p* < 0.05.

## 3. Results

### 3.1. Z-VAD Decreased the Metabolic Activity of Stimulated Macrophages but Not of Mesenchymal and Epithelial Cells

**Figure 1:**
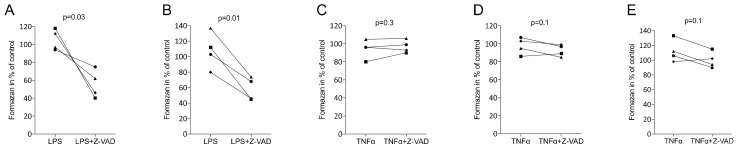
Z-VAD decreased the viability of macrophages but not of mesenchymal and oral epithelial cells. Cells were grown with and without Z-VAD in the presence of LPS or TNFα for 6 h and subjected to a classical MTT viability assay. Z-VAD decreased the viability of LPS-exposed RAW 264.7 cells (**A**) and primary macrophages (**B**). In contrast, Z-VAD showed no considerable impact on metabolic activity in ST2 bone marrow-derived stromal cells (**C**), gingival fibroblasts (**D**) or HSC2 oral squamous carcinoma cells (**E**). N = 4. Statistical analysis was based on a Friedman test, and *p*-values are indicated. Significance was set at *p* < 0.05.

**Figure 2:**
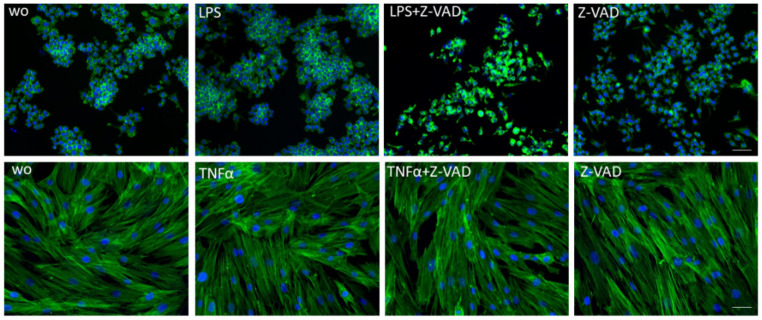
Phalloidin staining of filamentous actin in RAW 264.7 macrophages and gingival fibroblasts. Cells were grown with and without Z-VAD in the presence of LPS or TNFα for 6 h and subjected to phalloidin staining. Z-VAD caused shrinkage of the cells in RAW 264.7 macrophages but not in gingival fibroblasts. Scale bars represent 50 μm.

First, we tested the impact of Z-VAD on cell viability. Consistent with previous work [27,28], Z-VAD diminished the viability of LPS-exposed RAW 264.7 cells and macrophages in converting tetrazolium salt into formazan crystals (Figure 1A,B). In contrast, however, Z-VAD showed no considerable impact on the metabolic activity of ST2 bone marrow-derived stromal cells, gingival fibroblasts or HSC2 oral squamous carcinoma cells when exposed to TNFα (Figure 1C–E). Thus, the blocking of caspases with Z-VAD lowered the viability of macrophages, but not that of mesenchymal and epithelial cells, under inflammatory conditions. Consistently, phalloidin staining showed the shrinking of macrophages but not of gingival fibroblasts in the presence of Z-VAD and LPS or TNFα, respectively (Figure 2). We also confirmed that caspase-8 was active in macrophages and gingival fibroblasts but not increased by inflammation inducers [35] (data not shown).

### 3.2. Z-VAD Decreased the Inflammatory Response of LPS-Stimulated RAW 264.7 Cells and Primary Macrophages

**Figure 3:**
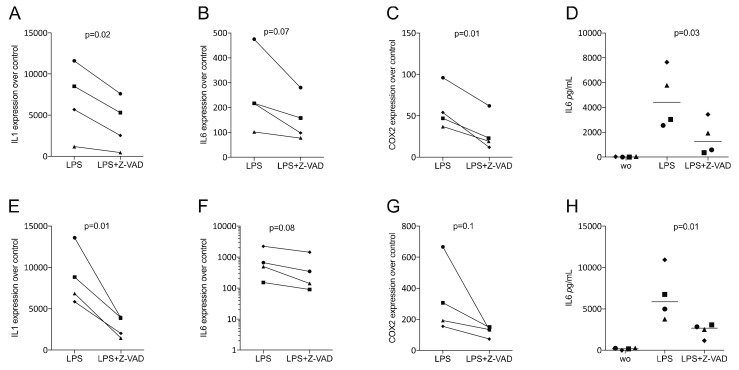
Z-VAD decreased the inflammatory response of LPS-stimulated macrophages. RAW 264.7 cells and primary macrophages were incubated with and without Z-VAD in the presence of 100 ng/mL LPS. The data indicate the x-fold changes of IL1, IL6 and COX2 gene expression compared to an unstimulated control and the concentration of IL6 in the supernatant in RAW 264.7 cells (**A**–**D**) and primary macrophages (**E**–**H**). Data points indicate independent experiments. N = 4. Statistical analysis was based on a paired *t*-test, and *p*-values are indicated. Significance was set at *p* < 0.05.

Considering that the impact of Z-VAD on the modulation of the expression of inflammatory cytokines has not been previously investigated [27,28] and that macrophages are target cells for a TLR-mediated inflammatory response, including periodontitis [36,37], we determined the capacity of Z-VAD to change the LPS-induced expression of inflammatory cytokines in murine macrophages. We show here that Z-VAD greatly reduced the capacity of LPS-exposed RAW 264.7 cells, as well as primary macrophages, to produce IL1, IL6 and COX2 (Figure 3).

### 3.3. Z-VAD Decreased the Inflammatory Response of TNFα-Stimulated ST2 Mesenchymal Cells and Gingival Fibroblasts

**Figure 4:**
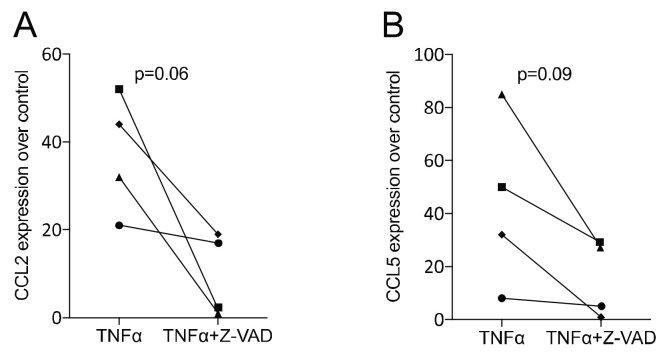
Z-VAD decreased the inflammatory response of TNFα-stimulated ST2 mesenchymal cells. ST2 cells were incubated with and without Z-VAD in the presence of 20 ng/mL TNFα. The data indicate the x-fold changes of (**A**) CCL2 and (**B**) CCL5 gene expression compared to an unstimulated control. Data points indicate independent experiments. N = 4. Statistical analysis was based on a paired *t*-test, and *p*-values are indicated. Significance was set at *p* < 0.05.

**Figure 5:**
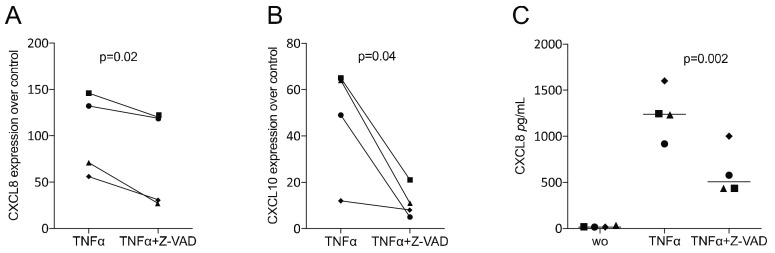
Z-VAD decreased the inflammatory response of TNFα-stimulated human gingival fibroblasts. Human gingival fibroblasts were incubated with and without Z-VAD in the presence of 20 ng/mL TNFα. The data indicate the x-fold changes of (**A**) CXCL8 and (**B**) CXCL10 gene expression compared to an unstimulated control and (**C**) the concentration of CXCL8 in the supernatant of gingival fibroblasts. N = 4. Statistical analysis was based on a paired *t*-test, and *p*-values are indicated. Significance was set at *p* < 0.05.

To verify whether the data observed in murine macrophages would translate into the mesenchymal lineage, we studied the impact of Z-VAD in a TNFα-exposed murine ST2 cell line. We found that Z-VAD significantly lowered the capacity of TNFα to drive the expression of the inflammatory cytokines CCL2 and CCL5 (Figure 4). Thus, blocking of caspases diminished the early inflammatory response of murine mesenchymal cells. To confirm that our findings observed with the ST2 murine mesenchymal cells would translate into the human system, we investigated Z-VAD in human gingival fibroblasts exposed to TNFα. Again, Z-VAD significantly diminished the expression of CXCL8 and CXCL10, which was driven by the presence of TNFα (Figure 5). Thus, in mouse mesenchymal cells and human gingival fibroblasts, the inhibition of caspases can reduce the TNFα-mediated inflammatory response.

### 3.4. Z-VAD Decreased the Inflammatory Response of TNFα-Stimulated HSC2 Oral Squamous Carcinoma Cells

**Figure 6:**
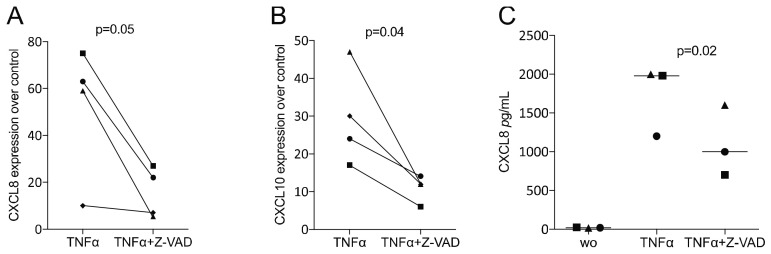
Z-VAD decreased the inflammatory response of TNFα-stimulated HSC2 oral squamous carcinoma cells. HSC2 cells were incubated with and without Z-VAD in the presence of 20 ng/mL TNFα. Data indicate the x-fold changes of (**A**) CXCL8 and (**B**) CXCL10 gene expression compared to an unstimulated control and (**C**) the concentration of CXCL8 in the supernatant of HSC2 cells. Statistical analysis was based on a paired *t*-test, and *p*-values are indicated. Significance was set at *p* < 0.05.

Finally, in order to include cells from the epithelial lineage, we provoked the expression of chemokines by TNFα in the presence of Z-VAD. Consistently, Z-VAD significantly reduced the TNFα-induced expression of CXCL8 and CXCL10 in HSC2 oral squamous carcinoma cells (Figure 6).

## 4. Discussion

Periodontitis is a chronic inflammatory disease, with virulence factors as the major but not exclusive drivers of the catabolic changes occurring through its hallmark effect, i.e., the loss of integrity of the periodontium [1,2]. It is the cytokines and chemokines produced by the local cells—macrophages, gingival fibroblasts and epithelial cells—that amplify the inflammatory process [3,4,7]. Periodontitis is functionally linked to caspases, which are not only molecular checkpoints of cell death. Caspases control the inflammatory response of cells and, consequently, tissue destruction apoptosis [21], pyroptosis [22,23] and necroptosis [24,25]. It is therefore not surprising that research attempts have been based on pharmacological blocking of caspases, including Z-VAD, aiming to reduce the inflammatory response of cells and mortality in mice [26,27,28] and the occurrence of renal failures [30,31], brain damage [32] and liver cirrhosis [33]. Our data fully support this concept and extend the knowledge gained with macrophages towards in vitro cellular models representing the periodontium. Our main findings are that the pan-caspase inhibitor Z-VAD considerably reduced the LPS- and TNFα-induced expression of cytokines and chemokines in murine macrophages, murine mesenchymal cells, human gingival fibroblasts and human oral epithelial cells.

Relating the findings observed with macrophages to those from other studies, our data are consistent with the previous findings that Z-VAD reduced the viability of murine macrophages in the presence of LPS [27,28]. Our findings further support observations according to which Z-VAD blocked LPS-induced secretion of inflammatory cytokines, including IL6, in bone marrow-derived and peritoneal macrophages in vitro [26]. In another in vivo study, using an endotoxic shock model, Z-VAD reduced the secretion of TNFα and inhibited LPS-induced CD86 expression in macrophages in the spleen and liver [26], further supporting our observation that macrophages are target cells for Z-VAD. However, this is not necessarily the case. In contrast to our work, Z-VAD has even been found to enhance TNFα release from LPS-exposed J774.1/JA-4 macrophages [38] and CXCL1 and CXCL2 release from LPS-exposed U937 macrophages [39]. Z-VAD further increased plasma cytokine and chemokine levels in mice after systemic TNFα administration [40]. Obviously, the impacts of Z-VAD on the modulation of an inflammatory response are heterogeneous, even within the macrophage cell lineage.

If we further relate our findings to those from other studies with respect to fibroblasts and epithelial cells, we are apparently publishing pioneering work on how Z-VAD affects cell viability and the inflammatory response to TNFα. Most research is focused on inflammation-induced cell viability and ROS production, for which Z-VAD was found to increase the sensitivity of L929 [41] and rat chondrocytes [42] to inflammatory clues. In our study, TNFα alone or in combination with Z-VAD had no negative impact on the metabolic activity of fibroblastic cells. However, Z-VAD significantly lowered the TNFα-induced expression of selected cytokines and chemokines in ST2 bone marrow stromal cells, gingival fibroblasts and HSC2 oral squamous carcinoma cells. These findings suggest that mesenchymal and epithelial cells can inhibit the capacity of Z-VAD to force cell death under inflammatory conditions.

The clinical relevance of the in vitro observations remains a matter of speculation, but they allow more general discussion about the role of caspases in periodontal health and disease. The clinical relevance is underlined by the caspase staining pattern in gingival biopsies of healthy and diseased tissue. Apoptosis [21], pyroptosis [22,23] and necroptosis [24,25] are active in chronic periodontitis and can therefore affect periodontal tissue integrity. However, it is unlikely that Z-VAD will become a pharmacological therapy to reduce local inflammation in periodontal tissues because a pan-caspase inhibitor targets all caspase-mediated events and might provoke unwanted side effects. The present research should be considered as a proof-of-principle study showing that cell types representing the periodontal structures are potential target cells for Z-VAD. The clinical use of caspase inhibitors has reached the level of phase II clinical trials; for instance, with psoriasis. The pharmacokinetic liabilities of these inhibitors, nevertheless, remain a concern [34].

The study limitations were that we could not attribute one particular caspase blocked by Z-VAD as responsible for the anti-inflammatory activity observed in periodontal cells. Future studies should therefore include more specific inhibitors—for instance, Gly-Phe β-naphthylamide, AP20187 and Z-IETD-FMK—to block caspase-8 and, thereby, open the necroptosis pathway [18]. Inhibitors raised against caspase-1, such as Z-YVAD-FMK, mulberroside A or chelidonic acid, can in turn inhibit the pyroptosis pathway [43]. Another limitation is that we do not know the reason why Z-VAD only reduced the expression of selected cytokines and chemokines, while IL1 and IL6 were not significantly suppressed by Z-VAD in ST2, gingival fibroblasts or HSC2 cells. Future studies should, therefore, implement RNAseq to understand which of the TNFα-induced target genes are functionally linked to caspase activity and identify those that are not reached by Z-VAD or other caspase inhibitors. Finally, it should be mentioned that in vitro models do not necessarily represent the complex situating of an inflamed periodontal site; hence, future research should focus on how local or systemic Z-VAD affect ligature-induced periodontitis in a preclinical model.

## 5. Conclusions

In summary, we show here that the pan-caspase inhibitor Z-VAD reduced the expression of selected cytokines and chemokines under in vitro conditions in a cell panel representing the periodontal tissues. The clinical translation of these findings remains challenging as pan-caspase inhibitors target all caspase-mediated events and might provoke unwanted side effects.

## Data Availability

The original contributions presented in the study are included in the article. Further inquiries can be directed to the corresponding author.
